# Uncovered p1 and p2 waves preceding the N3 vestibular evoked neurogenic potential in profound sensorineural hearing loss

**DOI:** 10.14814/phy2.14392

**Published:** 2020-03-13

**Authors:** Vicenç Pascual‐Rubio, Esther Domènech‐Vadillo, Paulina Cárdenas‐Escalante, Francesc X. Avilés‐Jurado, Anna Pellisé‐Guinjoan, Enric Figuerola‐Massana

**Affiliations:** ^1^ Department of Neurology and Clinical Neurophysiology Hospital Universitari Joan XXIII de Tarragona Tarragona Spain; ^2^ HJ23 Otorhinolaryngology Diseases Research Group‐IISPV Universitat Rovira i Virgili (URV) Tarragona Spain; ^3^ Department of Otorhinolaryngology Head‐Neck Surgery Hospital Universitari Joan XXIII de Tarragona Tarragona Spain; ^4^ Department of Otorhinolaryngology Head‐Neck Surgery Hospital Clínic de Barcelona Tarragona Spain

**Keywords:** N3 wave, profound sensorineural hearing loss, vestibular evoked neurogenic potential, vestibular system

## Abstract

**Background:**

The N3 wave is a vestibular evoked neurogenic potential detected in some patients with profound sensorineural hearing loss (PSNHL) during brainstem auditory evoked potential (BAEP) analysis. In 1998, Kato et al. mentioned two electropositive waves preceding N3, which we named p1‐p2, but no further description was given.

**Objective:**

We sought to demonstrate the reproducibility of these waves and hypothesize on their anatomic origin.

**Methods:**

We used two cohorts of patients with PSNHL. The first cohort comprised 10 patients with N3, allowing us to establish a new test with adequate electrophysiological conditions headed to detect p1‐p2 waves (PN3EP). The second cohort consisted of two groups: group A comprised 10 patients in whom N3 was not detected; and group B comprised 20 patients presenting N3. PN3EP was performed in both groups, of which 50% had cervical myogenic vestibular evoked potentials (cVEMPs).

**Results:**

Only group B presented p1‐p2. The PN3EP facilitated the identification of p1‐p2 over BAEP analysis, and their presence correlated well with cVEMPs.

**Conclusions:**

P1‐p2 may be covered due to inadequate BAEP setting conditions, and could be generated in the distal neural path that generates the N3 wave.

## Graphical abstract

In 1998 Kato T et al. mentioned two electropositive waves preceding N3 in patients with profound sensorineural hearing loss during brainstem auditory evoked potential (BAEP). We ideate a test, PN3EP, that allows a better characterization of these waves that could be distant potentials of the same vestibular pathway generating N3, acting as an “otolithic BAEP equivalent.”

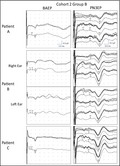

## 1 INTRODUCTION

The vestibular system, unlike other sensory systems such as vision or hearing, acts at a largely subconscious level in healthy individuals and it is not possible to accurately measure the vestibular sensation when the system is functioning correctly (Benson, 1982). Explorations focused on evaluating vestibular function in clinical practice are based principally on the reflexes that are generated, using, for example, nystagmography or cervical vestibular myogenic evoked potentials (cVEMPs). However, the study of reflexes has two basic limitations. The first limitation is that reflexes involve several anatomic structures out with the vestibular system, and their dysfunction can alter reflexes without necessarily implying a vestibular disorder. The second limitation is that some reflexes, such as the cervicovestibular reflex, require the patient´s active collaboration and they are difficult to accomplish in infants or noncooperative patients (Murofushi, 2009; Valente, 2011).

There are several vestibular evoked neurogenic potentials (VENPs) not based on reflexes that use the auditory capacity of the otolithic organs to study the vestibular system, including the N3, N5, N6 or P10 waveforms (Papathanasiou et al., 2005, 2010). Although less common in clinical practice, VENP testing provides direct information on the bioelectric signals transmitted by the vestibular nerve pathway, and is not dependent on extravestibular structures.

The N3 potential was first reported by Kato et al. (1998) as an electronegative wave with a latency of 3 milliseconds (ms) after auditory stimulation in some patients with profound sensorineural hearing loss (PSNHL) (Kato et al., 1998). Other authors prefer to use the term acoustically evoked short latency negative response or ASNR (Nong, Ura, & Noda, 2000). This evoked potential was first discovered by Cazals, Aran, Erre, Guilhaume, and Hawkins (1979), and several studies point to N3 as originating in the lower part of the brainstem by saccule activation in response to high intensity auditory stimuli (Burian, Gstoettner, & Zundritsch, 1989; Cazals, Aran, Erre, & Guilhaume, 1980; Cazals, Aran, Erre, Guilhaume, & Aurousseau, 1983; Cazals & Aurousseau, 1987; Murofushi, Iwasaki, Takai, & Takegoshi, 2005; Nong, Ura, Kyuna, Owa, & Noda, 2002; Ochi & Ohashi, 2001; Wit, Bleeker, & Segenhout, 1981). During brainstem auditory evoked potential (BAEP) in normal hearing patients, the vestibular response is masked by the greater amplitude and inverse polarity of the electropositive waves conforming the I‐V complex from the cochlear response. In the context of PSNHL, the waves of the I‐V complex disappear and are replaced by the vestibular evoked potential, N3, if the vestibular system is preserved. The criteria for N3 identification was established by Murofushi et al. (2005). N3 is detected in 11.9%–30% of patients with PSNHL during BAEP (Emara, 2010; Nong et al., 2000; Zagólski, 2007). Several studies compared cVEMP and N3, concluding that they have the same evoked potentials origin but different mechanism of generation (Emara, 2010; Jafari & Asad Malayeri, 2011; Murofushi et al., 2005; Nong et al., 2002; Ochi & Ohashi, 2001; Zagólski, 2008). N3 could have higher sensitivity and equivalent specificity to detect dysfunctions of the saccule nerve pathway than cVEMPs (Versino et al., 2007).

Kato et al. mentioned, without further details, two electropositive waveforms preceding N3 in the BAEP of a patient with SNHL, in the legend to Figure 3 of their study (Kato et al., 1998). To our knowledge, no other published studies have described these waveforms. Accordingly, the objective of the present study was to evaluate the prevalence of the two waves preceding N3, devise a new set of electrophysiological conditions for their optimal detection and description, and apply this new test to patients with PSNHL and compare it with cVEMP measurements. The study of these waves could extend our knowledge of the vestibular pathways and might help in the development of new diagnostic tools to test the vestibular system.

## 2 MATERIALS AND METHODS

### 2.1 Subjects

Two cohorts of patients with PSNHL were selected. The profound hearing loss was defined by an absence of auditory response to 90 dBnHL stimulus during BAEP. In all patients, the presence of conduction problem in the affected ears was ruled out by a thorough audiological examination, and several tests including tympanometry and nuclear magnetic resonance imaging. The first cohort was a retrospective cohort study comprised by 10 patients with PSNHL that presented N3 during BAEP. We used this cohort to determine the prevalence and morphologic description of the two electropositive waves preceding N3, which we have named p1 and p2 (“p” signifying electro positivity, and “1” or “2” for the approximate latency of appearance in ms).

The patients were recruited between 2009 and 2010 in the laboratory of Clinical Neurophysiology at Joan XXIII Hospital, Tarragona. The study group comprised six women and four men with ages ranging from 2 to 60 years. They contributed 14 ears (eight right ears and six left ears) with PSNHL and N3 present in BAEP. From these 10 patients, three had congenital PSNHL and contributed both ears to the study, seven were sudden PSNHL and only one of these patients contributed both ears to the study. The N3 waves of the selected patients complied with the first three criteria established by Murofushi et al. for the N3 definition (Murofushi et al., 2005): N3 should be reproducible; with a latency between 3 and 5 ms and an amplitude more than 0.05 µV from the initial deflection to the negative peak.

After the initial description, we established appropriate electrophysiological conditions (named the PN3EP test) oriented to specifically study the prevalence and morphology of p1 and p2.

A second cohort of patients was from a prospective and descriptive research study conducted at Joan XXII Hospital between 2010 and 2013. This cohort comprised two groups of patients with PSNHL. Group A included six women and four men, with ages ranging from 6 months to 63 years. They contributed 11 ears (6 right ears and 5 left) with the absence of N3 in BAEP. From these 10 patients, one had congenital PSNHL and contributed both ears to the study, six were sudden PSNHL and contributed with one ear, and three were PSNHL secondary to an infectious process and contributed with one ear. From the group A patients, five were submitted to cVEMP test. Group B comprised 20 patients, 11 women and nine men, with ages ranging from 2 months to 70 years. They contributed 25 ears (13 right ears and 12 left ears) with the presence of N3 in BAEP. Of these patients, four had congenital PSNHL and contributed both ears, 15 were sudden PSNHL and contributed with one ear and in one case with two ears, and one had PSNHL secondary a traumatic head injury and contributed with one ear to the study. The N3 of the selected patients complied with N3 definition criteria (Toshihisa Murofushi et al., 2005). From group B patients, 10 had the cVEMP test performed.

The study was approved by the ethics committee of the *Hospital Universitari Joan XXIII, Tarragona*. The study was explained to all patients and, in the case of pediatric patients, to parents or legal guardians. In all cases, they provided their informed consent.

## 3 MATERIAL

The BAEP and PN3EP were performed using an evoked potential recording system (Synergy, CareFusion). The system included an analogical/digital converter with a 16‐bit resolution for acquired data. The auditory stimulation was presented through adaptable earphones according to recommendations for the practice of clinical neurophysiology, ISO 9000, IEC 601. Responses were recorded by applying surface Ag/AgCl electrodes of 10 mm diameter impregnated with an electro‐conductive gel (Spectra 360, Parker Laboratories Inc.) on the scalp. The skin was cleaned with an abrasive gel before attaching the electrodes. The cVEMP recordings were obtained using two different devices. The evoked potential recording system (Synergy) used to record the BAEP, but adapted to record cVEMP, and an evoked recording system (Bio‐logic, Natus Medical Incorporated), which has a rectifier program included.

### 3.1 Recording and stimuli conditions

Brainstem auditory evoked potential was performed locating the electrodes according to the 10:20 system (International Federation of Societies for EEG and Clinical Neurophysiology). Two channels were recorded with the active electrodes located over the skin region of the mastoid process and both referenced to the vertex electrode (Cz). A stimulation of 90 dBnHL with rarefaction clicks of 0.1 ms duration was used in healthy ears and the intensity was increased to 105 dBnHL in PSNHL ears. The stimuli repetition rate was 11 Hz and the contralateral white noise was applied with an intensity of 30 dBnHL lower than the ipsilateral stimuli. The band pass was 0.1 Hz–3 kHz. The maximum impedance accepted was 5 KΩ. A total of 1,000 trials were averaged over 15 ms sweep duration with a sensitivity of 500 nV. Reject amplitude artifact interval was 40 µV. In the PSNHL ears, the waves were quantified by calculating an average of 1,000 responses that was repeated three times to ensure reproducibility. The response was calculated by performing a “global” average of all 3,000 responses to allow a better identification of p1 and p2. The recording of the electrode located in the ipsilateral mastoid and referred to the vertex was presented.

For the PN3EP, the proposed setting of electrophysiological conditions was applied using the same recording montage as for BAEP, but adding another channel with an active electrode located over the skin region of the seventh cervical vertebra spine (EvC7) and referenced to the vertex (Cz). Stimuli (clicks) were produced by delivering square waves of 0.1 ms duration, with alternating polarity, an intensity of 105 dBnHL and a stimuli repetition rate of 20 Hz. The masking condition of the contralateral ear was applied with white noise of 75 dBnHL. The band pass was broadened to an interval of 0.1 Hz–10 kHz; the maximum impedance accepted was 2 kΩ. An average of 5,000 responses was repeated four times to ensure reproducibility. The global average of 20,000 sweeps were recorded over a 5‐ms sweep with a sensitivity of 200 nV. Reject amplitude artifact interval was 20 µV.

The cVEMP electrodes were placed over the sternocleidomastoid muscle (SCM) muscle belly (active), medial clavicle (reference), and sternum (earth). Subjects were stimulated in both ears simultaneously while they performed a maximal SCM contraction in supine position (Wang & Young, 2003). The stimulus was an auditory click of 0.1 ms duration using insert earphones and delivered at a rate of 5 Hz for 150 repetitions per trial. Stimulus intensity was established at 95 dBnHL. SCM muscle activity was recorded bilaterally; the signal was recorded over a 50 ms duration acquisition and filtered with band pass of 10 Hz–1.5 kHz. The negative potentials at the active electrodes were displayed as upward deflections.

## 4 ANALYSIS OF THE DATA

We proposed to describe the p1 and p2 waves by their latency, amplitude and duration. Latency was measured at the beginning of the rising phase of the wave. The amplitude was measured from the beginning of the ascending phase to the highest peak of the wave; and the duration was measured from the beginning of the ascending phase to the end of the descending phase. The selected description variables were arithmetic mean, standard deviation, and a confidence interval of 95% for mean. The medians were used for ordering descriptions. For the means comparison, the normality of the distribution was checked by Kolmorogov‐Smirnov test and Student’s *t* test was used for analysis. Statistical analysis was performed using IBM SPSS 20.0 for Windows (SPSS).

## 5 RESULTS

### 5.1 p1 and p2 identification and prevalence

**FIGURE 1:**
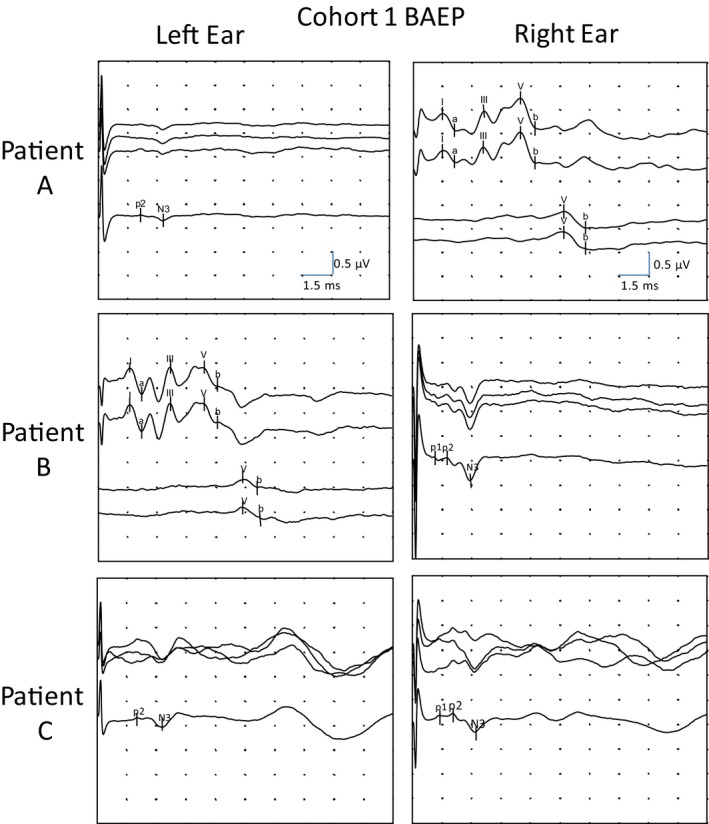
Brainstem auditory evoked potential (BAEP) from three patients of cohort1 (left ear stimulated on the left and right ear on the right). *BAEP conditions*: Sweep: 15 ms. Sensitivity: 500 nV/division. Band pass 0.1Hz–3 kHz. Click of 0.1 ms duration. The recording channel corresponds to the electrode located in the ipsilateral mastoid and referenced to the vertex. Healthy ears: stimulus intensity of 90 and 30 dBnHL. Average of 1,000 responses that was repeated two times to ensure reproducibility. The global average with the marks was of 2,000 responses. Profound sensorineural hearing loss (PSNHL): Stimulus of 105 dBnHL. Average of 1,000 responses that was repeated three times to ensure reproducibility. The global average with the marks was of 3,000 responses. Patient A was a 34‐year‐old woman with left sudden PSNHL. Patient B was a 37‐year‐old woman with right sudden PSNHL. Patient C was a 41‐year‐old man bilateral congenital PSNHL

All reproducible evoked potentials preceding N3 were selected. From the first cohort, 14 ears with PSNHL, 78% (*n* = 11) presented reproducible waves, p1 or p2, preceding N3. Of those, 18% (*n* = 2) presented both p1 and p2. The polarity of these waves in all cases was interpreted as electropositive. We selected two groups of waves: the first group had five waves with latency intervals ranging from 1.1 to 1.4 ms and were considered the p1 type. The second group had eight waves ranging from 1.7 to 2.2 ms and were considered the p2 type (Figure 1). The p1 and p2 waves represented a prevalence of 35.7% and 57.1%, respectively. The mean amplitude of p1 was 0.04 ± 0.02 µV. The p2 group had larger mean amplitude than the p1 group, but with a greater variability (0.07 ± 0.04µV). The duration of p1 and p2 was very similar and less variable than the amplitude (0.41 ± 0.11 ms and 0.54 ± 0.08 ms for p1 and p2, respectively; Table 1).

**TABLE 1:** Preliminary study: p1, p2, and N3 description from 10 patients with PSNHL during BAEP ipsilateral channel. Prevalence in %. Mean and standard deviation (*SD*) of duration and latency to peak, in milliseconds; amplitude to peak in microvolts

Wave	Prevalence (%)	Latency (ms)	Duration (ms)	Amplitude (µV)
p1	35.7	1.25 (*SD* 0.14)	0.41 (*SD* 0.11)	0.04 (*SD* 0.02)
p2	57.1	2.01 (*SD* 0.12)	0.54 (*SD* 0.08)	0.07 (*SD* 0.04)
N3	100	3.17 (*SD* 0.14)	1.46 (*SD* 0.41)	0.22 (*SD* 0.15)

Abbreviations: BAEP, Brainstem auditory evoked potential; PSNHL, profound sensorineural hearing loss.

### 5.2 PN3EP results

**FIGURE 2:**
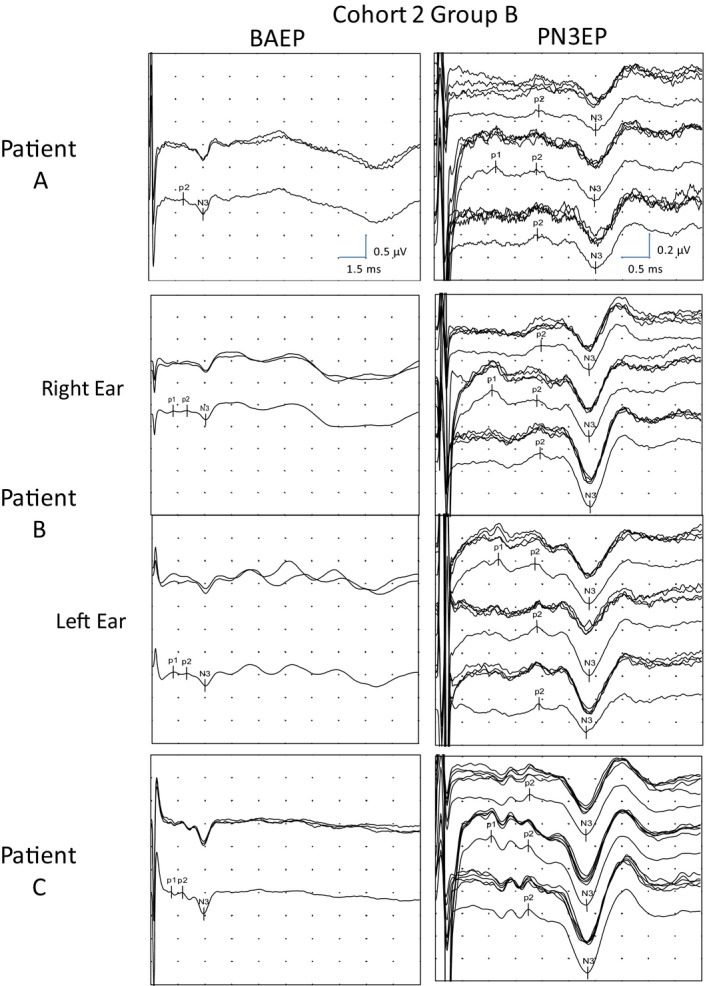
Brainstem auditory evoked potential (BAEP; left image) and PN3EP (right image) from three patients of cohort 2 group B. *BAEP conditions*: Sweep: 15 ms. Sensitivity: 500 nV/division. Stimulus 105 dBnHL at 11 Hz. Average of 1,000 responses that was repeated three times to ensure reproducibility. The global average with the marks was of 3,000 responses. The recording channel corresponds to the electrode located in the ipsilateral mastoid and referenced to the vertex. *PN3EP conditions*: Sweep: 5 ms. Sensitivity: 200 nV/division. Stimulus of 105 dBnHL at 20 Hz. Average of 5,000 responses that was repeated four times to ensure reproducibility. The global average with the marks was of 20,000 responses. Three recording channels are used. The first between the left mastoid and the vertex; the second between the right mastoid and the vertex; and the last between the skin region of the seventh cervical vertebra spine (EvC7) and referenced to the vertex (Cz). Patient A was a 39‐year‐old man with right sudden profound sensorineural hearing loss (PSNHL). Patient B was a 34‐year‐old man with bilateral congenital PSNHL. The two upper images of this patient are from the right ear and the lower two from the left. Patient C was a 37‐year‐old woman with right sudden PSNHL. The p1‐p2 waves appear between the large stimulus artifact (in the first 1 ms of the recording) and the N3 wave. Patient C shows a biphasic p2 potential

**FIGURE 3:**
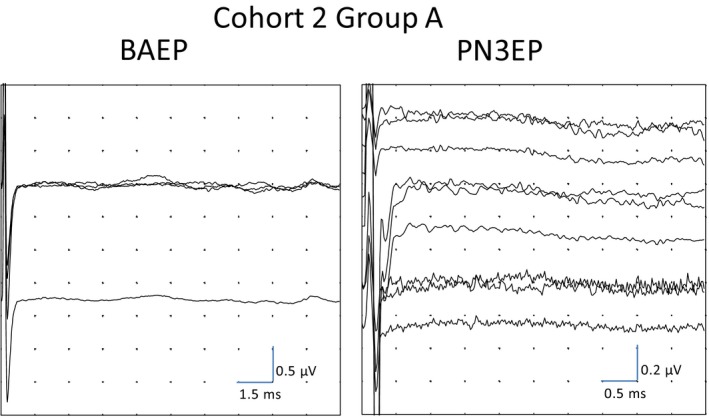
Brainstem auditory evoked potential (BAEP; left image) and PN3EP (right image) from cohort 2, group A 60‐year‐old woman with right sudden profound sensorineural hearing loss. No reproducible wave is observed in the BAEP or the PN3EP

**FIGURE 4:**
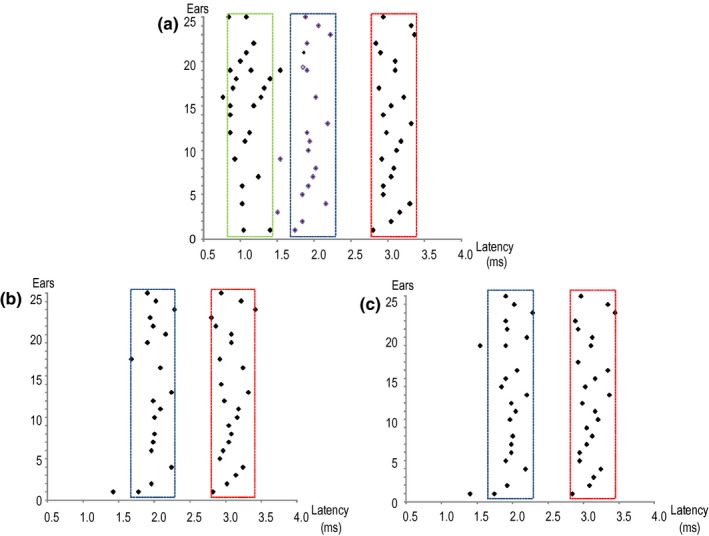
Distribution of the PN3EP in the three recording channels determined by visual inspection. (a) Ipsilateral mastoid process referenced to the vertex; (b) contralateral mastoid process referenced to the vertex; (c) seventh cervical vertebra spine referenced to the vertex. On the ipsilateral channel the waves from the global 20,000 average are organized in three columns: around 1(p1, green box), 2 (p2, blue box) and 3 ms (N3, red box). Whereas on the contralateral and cervical channels the waves are grouped in two columns: p2 (blue box), and N3 (red box)

**FIGURE 5:**
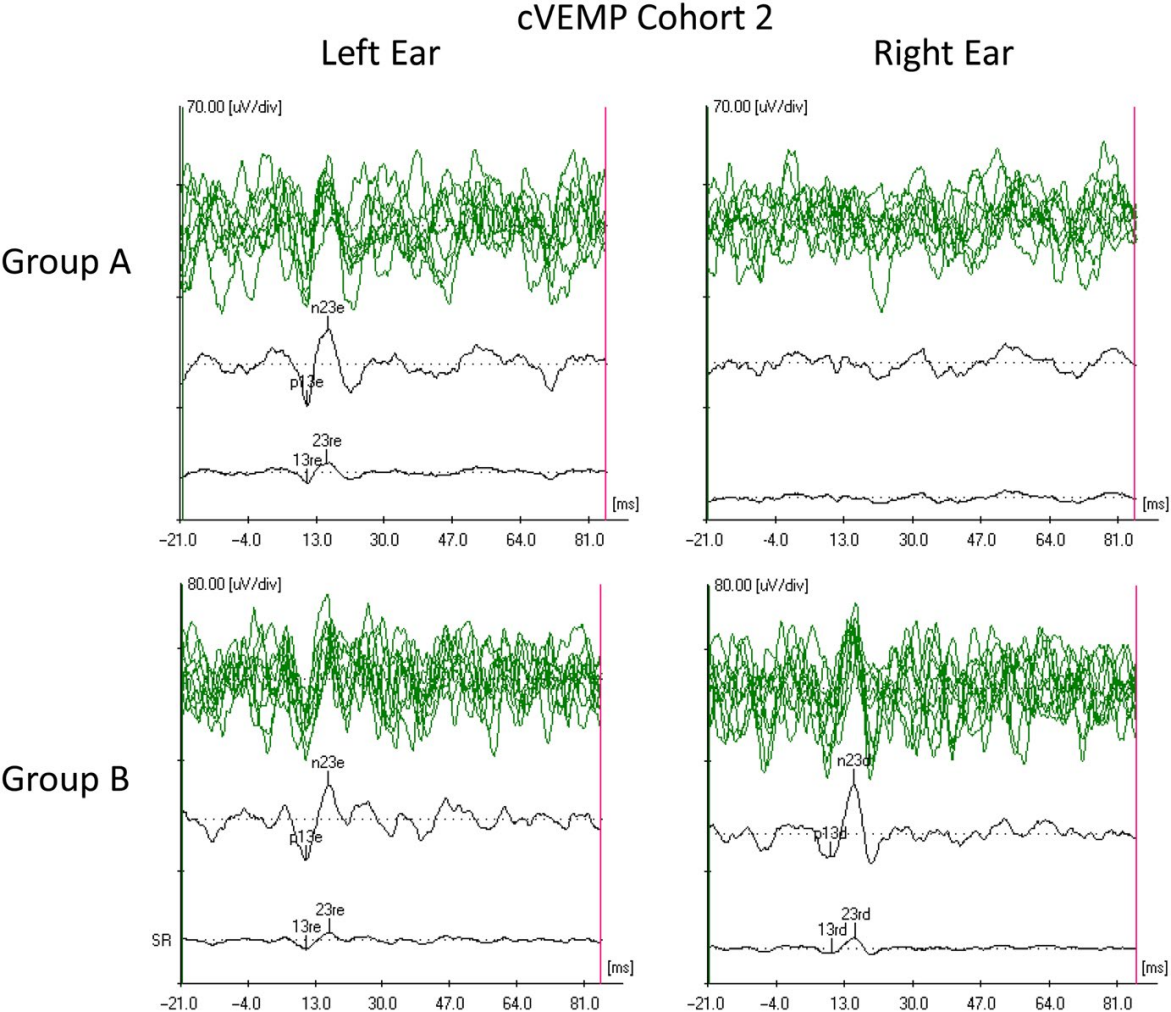
Cervical myogenic vestibular evoked potentials from two patients of cohort 2 (left ear stimulated on the left and right ear on the right). A 95 dBnHL at 5 Hz stimulus was applied to both ears simultaneously while recording the SCM in maximal contraction. We show several averages of 150 stimulations with a total average, in which we placed the markers p13‐p23 (“e” meaning left, and “d” meaning right), and a rectification of the response: 13r‐23r. On top, a 35‐year‐old woman from group A, with right sudden profound sensorineural hearing loss (PSNHL). No evoked potential is observed from the right vestibulocervical reflex. Below, a 16‐year‐old woman with right sudden PSNHL from group B. Normality of the response

The p1 and/or p2 waves were detected in the majority of patients from group B (Figure 2), whereas none of the patients from group A presented p1 or p2 waves (Figure 3). The latencies from each global average waveform were identified and collocated in a graphic for visual detection of the distribution of the waves in each channel (Figure 4). The ipsilateral channel were distributed in three groups around the latencies 1, 2, and 3 ms, and were grouped as p1, p2, and N3 taking in account the first cohort latency description. The intervals were established from 0.5 to 1.5 ms for p1, and from 1.5 to 2.5 ms for p2. In the contralateral and cervical channels, p1 was not detected. Biphasic potentials were detected for p1 (35%) and p2 (8.8%). We selected the longest latency from the two peaks because they showed the higher amplitude. Prevalence, latencies, interval latencies, duration, and amplitude parameters were analyzed for p1, p2, and N3 from each channel and are shown in Table 2. No statistical differences were detected between the electrophysiological characteristics of p2 waves from contralateral and cervical PN3EP channels (*p* < .05). In one patient, no p1, p2 or N3 was detected during the PN3EP. This patient was not included in the statistical description of p1, p2, and N3 waves. No evoked response was detected in the cVEMP from group A patients, whereas all 10 patients from group B presented p13 and n23 evoked potentials with normal values (Figure 5).

**TABLE 2:** Description of Group B electrophysiological parameters. P1, p2 and N3 during brainstem auditory evoked potential (BAEP) and PN3EP application in relation of each channel. Prevalence in %. Mean and standard deviation (*SD*) of duration and latency to peak, in milliseconds; amplitude to peak in microvolts

BAEP	Ipsilateral	Wave	Prevalence (%)	Latency (ms)	Amplitude (µV)	Duration (ms)	Latency interval (ms)
		p1	41.7	1.23 (*SD* 0.20)	0.07 (*SD* 0.04)	0.50 (*SD* 0.15)	
		p2	58.3	1.97 (*SD* 0.18)	0.06 (*SD* 0.03)	0.56 (*SD* 0.06)	
		N3	100	3.17 (*SD* 0.17)	0.38 (*SD* 0.39)	1.39 (*SD* 0.54)	
		p1‐p2					0.74 (*SD* 0.11)
		p2‐N3					1.20 (*SD* 0.23)
		p1‐N3					1.88 (*SD* 0.16)
PN3EP	Ipsilateral	p1	70.8	1.11 (*SD* 0.14)	0.10 (*SD* 0.07)	0.45 (SD0.13)	
		p2	79.2	1.92 (*SD* 0.18)	0.08 (*SD* 0.03)	0.50 (*SD* 0.08)	
		N3	95.8	3.06 (*SD* 0.16)	0.26 (*SD* 0.13)	1.21 (*SD* 0.18)	
		p1‐p2					0.80 (*SD* 0.13)
		p2‐N3					1.16 (*SD* 0.16)
		p1‐N3					1.91 (*SD* 0.18)
	Contralateral	p1	0	‐	‐	‐	
		p2	79.2	2.01 (*SD* 0.15)	0.04 (*SD* 0.02)	0.52 (*SD* 0.10)	
		N3	91.7	3.07 (*SD* 0.16)	0.20 (*SD* 0.11)	1.17 (*SD* 0.19)	1.15 (*SD* 0.16)
		p2‐N3					
	Cervical	p1	0	‐	‐	‐	
		p2	8.3	1.99 (*SD* 0.13)	0.06 (*SD* 0.03)	0.49 (*SD* 0.09)	
		N3	95.8	3.10 (*SD* 0.16)	0.31 (*SD* 0.17)	1.33 (*SD* 0.20)	
		p2‐N3					1.12 (*SD* 0.11)

## 6 DISCUSSION

Because of their small amplitude and short latency and duration, p1 and p2 waves are probably covered up during BAEP in patients with PSNHL presenting N3. The initial description of those waves from the first cohort allowed us to propose a new set of electrophysiological conditions to obtain a better characterization.

To minimize the stimulus artifact close to p1, we used clicks of square waves of 0.1 ms duration. According to Kato et al., polarity and stimuli repetition rate equal to or below 40 Hz does not modify N3 significantly (Kato et al., 1998). We established alternating polarity to 20 Hz stimuli, which is the maximum repetition rate allowed on our device. The stimulus intensity for the N3 generation has been established at 95–100 dBnHL by several authors (Kumar, Bhat, & Guttedar, 2011; Toshihisa Murofushi et al., 2005; Nong et al., 2002; Ochi & Ohashi, 2001), and we choose 105 dBnHL to ensure activation of the otolithic organs. Although acknowledged as a high intensity that is greater than accepted safe levels, we believe that it is safe for the patient because the duration is short and the stimulus is applied only to ears of patients with PSNHL. After hearing stimulation at 105 dBnHL with PSNHL, no patient showed any discomfort or symptoms added to the pretest situation. In our opinion, the masking conditions of the contralateral ear do not need to be changed. We added a third channel, EvC7, acting as a far‐field potential that aims to record the maximum information possible despite being more influenced by the electrical environment noise. The low pass filter frequency used during BAEP could affect p1‐p2 waves significantly because a wave of 0.4 ms duration corresponds to a frequency of 2,500 Hz and is too close to the established 3,000 Hz high frequency cut‐off. We therefore propose to increase the filter interval from 100 to 10,000 Hz. We averaged 20,000 responses to compensate the high environmental noise that is expected because of the low amplitude of the evoked response. This increment in the number of responses lengthens the duration of the test to a total of 10 min for each ear studied. In order to increase the bioelectric signal resolution of p1, p2 the sweep is reduced to 5 ms and the sensitivity increased to 200 nV/division. The interval of amplitude of the artifact rejection is limited to ±20 µV to reject the bioelectrical signal not evoked by the acoustic stimulus. The electrode impedance in contact with the skin should be less than 2 kΩ to optimize the bioelectrical signal. As occurs with BAEP, we would elect not to use the AC current filter in order to avoid interference in the wave morphology.

All patients in group B presented p1 or p2 in contrast to the absence of both waves in group A patients. In addition, none of the group A subjects presented a response during cVEMP, unlike those in group B, suggesting that p1 and p2 are consistent with N3 and are probably evoked potentials of vestibular origin (Figure 5). We show that the prevalence of p1 and p2 is higher in PN3EP ipsilateral channel as compared with BAEP measures, supporting the idea that the proposed electrophysiological conditions are more appropriate for p1 and p2 characterization. An explanation for the lower prevalence of p1 compared with p2 could be because the short p1 latency positions it very close to the stimulation artifact, which could occasionally mask it. All N3 values obtained in our study were similar to those described in the literature (Emara, 2010; Kato et al., 1998; Murofushi et al., 2005; Nong et al., 2000; Nong et al., 2002; Ochi & Ohashi, 2001; Versino et al., 2007; Zagólski, 2008).

Despite the difference in prevalence, the morphology of p1, p2, and N3 were similar between PN3EP and BAEP. This was expected because the type of stimulus and the recording localization of the electrodes were not changed in the PN3EP set conditions. Therefore, comparing with BAEP, the PN3EP have the advantage of a better detection without changing the p1, p2, and N3 wave morphologies and allows a more specific vestibular evaluation.

The high prevalence of p1 and p2 biphasic potentials is striking although the double peak is a frequent phenomenon in the analysis of evoked potentials, for instance BAEP wave II (Ananthanarayan & Durrant, 1991; Figure 2). Biphasic potentials are attributed to the bioelectric vector orientation regarding the recording electrodes or the presence of multiple electrical generators of the evoked potential (Kimura et al., 1986; Stegeman, Dumitru, King, & Roeleveld, 1997). To investigate those options, it might be necessary to perform other studies using different recording and stimuli configurations.

The fact that contralateral and cervical channels are not significantly different suggests that PN3EP could be performed with the same montage used during BAEP. This would facilitate the PN3EP application as a complementary test to evaluate the vestibular system when BAEP is not obtained. With the test duration of 20 min, PN3EP could facilitate an early detection of vestibular dysfunction and allow for the implementation of early programs for equilibrium application in infants.

The description of p1 and p2 waves allows us to hypothesize on their anatomic origin, although we are aware of the limitations involving the interpretation of evoked potentials since they are usually the result of complex summations of different bioelectric vectors. Since p1 and p2 waves have similar and stable amplitude, duration and interval latencies, they are probably evoked potentials from different points of the same neural pathway that generates N3. P1 appeared only in the ipsilateral channel and p2 appeared in all three channels. This allows us to propose that p1 could originate in the most distal part of the vestibular inferior nerve or in the Scarpa ganglia, and p2 in the proximal part of the vestibular inferior nerve, at the entrance of the vestibular nerve in the brainstem. Similar observations have been reported for BAEP wave I and II (Møller, Jannetta, Bennett, & Møller, 1981; Møller, Jannetta, & Sekhar, 1988). That the amplitude of p1 and p2 is lower than that of N3 (~25%) could be explained by the central amplification phenomenon that is usually seen during evoked potential analysis, where the central nervous responses are enhanced over the peripheral responses. For instance, wave I of neural peripheral origin is usually lower in amplitude than BAEP waves III and V.

Both p1 and p2 are electropositive evoked potentials so they probably have the similar vector orientation as BAEP waves I and II. This agrees with the similar nerve trajectory of cochlear nerve and inferior vestibular fascicle. By contrast, the N3 is electronegative differing from BAEP waves III and V, likely because of the descendent course of the vestibulospinal tract (Emara, 2010; Murofushi et al., 2005; Sato, Imagawa, Isu, & Uchino, 1997).

According to our hypothesis, the p1‐p2 interval could be the conduction time of the inferior vestibular fascicle and the p2‐N3 interval could be the neural conduction time from the entrance to the brainstem before their descendent fibers. As shown, the p1‐p2 interval latencies are shorter than p2‐N3 and this is likely because the latter involves one neuron synapse in the vestibular nuclei.

Although waves p1, p2, and N3 from PN3EP cannot be compared with BAEP waves I, II, and III because of the different sensorial activation mechanism, we can imagine that the lower amplitude of p1 and p2 potentials compared with wave I and II could be in relation to the smaller number of axonal fibers participating in the nerve conduction: 30,000 axons from the auditory nerves and 20,000 axons from the vestibular nerves (Moriyama, Itoh, Shimada, & Otsuka, 2007; Spoendlin & Schrott, 1989). Moreover, from these 20,000 fibers, only a small portion of the vestibular fibers has the capacity of response to sound (Cazals et al., 1983; Cazals & Aurousseau, 1987).

We believe that PN3EP could act as an “otolithic BAEP equivalent” with the same capacity of neural path segmentation on intervals of latencies. This would allow us to detect focal lesions in the peripheral vestibular pathway. The limitation is that it can only be applied in PSNHL patients, although there are studies proposing forms to achieve N3 in normal hearing individuals (Murofushi et al., 2005). If the technical part could be improved, and if it were possible to stimulate at 40 Hz, PN3EP could decrease the time spent obtaining responses and it could be proposed as an intraoperative neurophysiologic monitoring test during surgery with an associated risk of affecting the vestibular nerve system. For instance, during cochlear implantation, where the saccule could be affected during traumatic insertion of the prosthesis because of the cochlea proximity (Handzel, Burgess, & Nadol, 2006; Krause, Louza, Wechtenbruch, & Gürkov, 2010; Melvin, Della Santina, Carey, & Migliaccio, 2009; Welling, Hinojosa, Gantz, & Lee, 1993). Further, the increasing use of bilateral cochlear implants makes it necessary to develop new tests to reduce the risk of vestibular lesion (Melvin et al., 2009).

This study has several limitations. As the first cohort study is retrospective, the fourth criteria of Murofushi's N3 definition about the N3 disappearance when the external auditory canal is occluded, cannot be applied in cohort 1. To avoid the selection of nonvestibular components, only the evoked potentials disappearing below 90 dBnHL are collected. We did not evaluate the possibility that the otolithic system was partially affected. Nevertheless, with N3 present in the BAEP, we know that the saccule function is, at least in part, preserved so we could carry out an approximate description of p1 and p2. Only 50% of patient's in the first cohort had the cVEMPs done. The same proportion was achieved in the second cohort although being a prospective study. This is because we included infants in the study and we are not used to performing them cVEMPs. In addition, in group B of the second cohort, there were patients over 65 years of age in whom cVEMP test was not recommended. Of note, in two adult cases, we lost contact before performing cVEMP. Another limitation is that no maximum time interval between tests (BEAP, PN3EP, and cVEMP) are set. This fact prevents the evaluation of the effect of dynamic process of alteration or recuperation of the vestibular system, but does allow us to include patients with congenital PSNHL. The patient excluded from the statistical analysis was a 56‐year‐old woman with sudden left PSNHL who did not express evoked potentials during PN3EP and cVEMP. We attribute this finding to a dynamic process affecting the vestibular system as BAEP was performed several years earlier and she presented an important deterioration in her vestibular function in this period.

In conclusion, the two electropositive waves preceding N3 described by Kato T et al. appear in a consistent manner with N3. PN3EP allows us to better identify these waves, which we have named p1 and p2. Their electrophysiological characteristics and relationship with cVEMP appearance lead us to hypothesize that these waves could be distal potentials of the same vestibular pathway generating N3, acting as an “otolithic BAEP equivalent.” Although this is a pilot study, the current investigations will help clinicians to test if the hearing loss is accompanied by vestibular impairment.
